# Barriers and facilitators of weight management: Perspectives of the urban poor in Accra, Ghana

**DOI:** 10.1371/journal.pone.0272274

**Published:** 2022-08-08

**Authors:** Grace Frempong Afrifa-Anane, Delali Margaret Badasu, Samuel Nii Ardey Codjoe, John Kwasi Anarfi

**Affiliations:** 1 Department of Environment and Public Health, University of Environment and Sustainable Development, PMB, Somanya, Ghana; 2 Regional Institute for Population Studies, University of Ghana, Legon-Accra, Ghana; Public Library of Science, UNITED KINGDOM

## Abstract

**Background:**

Obesity is rising in sub-Saharan Africa and globally, and is a highly significant public health problem that needs urgent attention. To reduce the obesity prevalence levels and associated challenges, public health interventions highlight healthy eating and increased physical activity, which are key elements for weight management.

**Aim:**

This study explored perceived factors that hinder or facilitate weight management in an urban poor context in Accra, Ghana.

**Methods:**

A cross-sectional qualitative data was obtained from eight focus group discussions (FGDs) conducted among community members. The FGDs were audio recorded, transcribed, and analysed using thematic analysis.

**Results:**

The findings indicate that weight management is mainly inhibited by the perception that healthy foods are expensive and not satisfying, laziness, lack of time to engage in physical activity, and social representation of being fat or slim. On the contrary, personal decision to manage weight and social support facilitated engagement in weight management.

**Conclusion:**

The findings indicate that weight management is influenced by individual, community, and structural factors. These findings have programmatic implications in terms of health education strategies and messaging.

## Introduction

The prevalence of obesity is increasing globally, and even the poor regions of low and middle-income countries (LMICs) have recorded high prevalence rates. Sub-Saharan Africa (SSA), particularly in urban settings, has also observed an increasing trend [1–3], and some studies have indicated that obesity among adults is increasing in Ghana [4, 5]. Obesity poses a global health challenge as it undermines health and development and increases the risks of cardiovascular diseases (CVDs), diabetes and cancers [6, 7].

Therefore, prevention and management of obesity are key health priorities worldwide. Thus, public health interventions emphasize lifestyle changes such as increasing physical activity and eating healthy diets to attain a healthy body weight [8]. Although maintaining a healthy weight is essential for overall health, engaging in a healthy lifestyle requires motivational skills and attitudinal change [9]. Some studies have been undertaken elsewhere on the issue, but very few have been conducted in sub-Saharan Africa. For example, a study by Aziz et al. [10] among Malaysian overweight and obese housewives reported that lack of time and lack of support from family, friends and the environment were the key barriers to weight management.

Other studies have identified the following barriers to weight management: lack of time, lack of social support, lack of money to access healthy food, difficulty in changing diets, limited availability of healthy foods, and lack of physical activity spaces [11–13]. However, social support from family and friends, knowledge about the benefits of weight management, self-motivation, and health concerns have been reported to facilitate weight management [10, 14].

In sub-Saharan Africa, few studies have focused on perceptions of weight management. For example, Draper et al.’s [15] study on black South African women in a low resourced context indicates that although black women knew the benefits of healthy eating and exercise, factors such as higher cost of healthier foods, food insecurity, and lack of facilities and equipment for exercise programmes created a barrier to a healthy lifestyle among them. This study, therefore, intends to add to the limited literature on perceptions of weight management in sub-Saharan Africa by exploring community perceptions of barriers to and facilitators of weight management in low-income communities in Accra, the capital city of Ghana, using qualitative research methodology.

## Materials and methods

### The study context

The study was conducted in Ussher Town and James Town in Accra, the capital city of Ghana.

These two communities are contiguous and constitute the area called Ga Mashie, meaning indigenous Ga. The primary language spoken by these people is Ga, and the dominant economic activity is fishing and fish mongering. Ga Mashie is one of the oldest communities in Accra and a typical representation of urban poor communities in Accra. It is characterised by poor housing structures, sanitation, limited health care access, and low educational status [16]. In addition, the urban economic activity dynamics have led to sedentary activities in these communities. Thus, some earlier studies have reported evidence of a high incidence of obesity and chronic diseases in Ga Mashie [17, 18].

### Study design

The study is a part of a larger study (titled: Body size estimation and weight management behaviours) conducted in Accra, Ghana. The main study aimed to examine the influence of body size estimation on weight management behaviours in two urban poor communities in Accra, Ghana. It was cross-sectional data gathered with quantitative and qualitative instruments. The convergent parallel mixed method was adopted for the data collection. This design enabled the researcher to collect both quantitative and qualitative data at approximately the same time and incorporate the information to interpret the overall findings. The present study explored issues on factors that influence engagement in weight management behaviours using qualitative research methods. Qualitative research methods help to gain deeper insight into the participants’ perspectives. Furthermore, exploring the experiences and views of the participants can contribute to the development of appropriate public health strategies to help decrease the incidence of obesity and related chronic non-communicable diseases among these vulnerable groups. An interview guide was used to conduct eight FGDs (four from each community) from November 10 to December 1, 2016. The interview guide covered socio-demographic characteristics, community perceptions and preferences of body size, benefits of weight management, and barriers to and facilitators of weight management practices (S1 File). Anthropometric measures (height and weight) of respondents were also taken. Height measurements were obtained using a measuring tape (5 M/16FT measuring tape) in centimeters (cm) after the removal of slippers or shoes, and a weighing scale (Seca Scale with a maximum measurement of 150 kg) was used to take participants’ weights in kilograms (kg). BMI was calculated by dividing the weight (kg) of the participants by their height in meters squared (m^2^). They were classified as being underweight (BMI<18.5 kg/m^2^), normal weight (BMI from 18.5kg/m2 to 24.9 kg/m^2^), overweight (BMI from 25 kg/m2 to 29.9 kg/m^2^), and obese (BMI ≥ 30 kg/m^2^) according to the World Health Organization’s (WHO) cut-off points [19].

The focus group discussions (FGDs) were segmented by age group (younger adults-18-35 years and older adults-36 years and above), sex (males and females), and locality (James Town and Ussher Town) to ensure homogeneity in the sample and provide the opportunity for members to express themselves and contribute to the discussion. Three interviewers (the first author and two trained research assistants) conducted the group discussions. The FGDs were conducted in two local languages (Ga and Twi) and were held in a quiet environment within the community. A FGD session took approximately 40 minutes and was audio-recorded with participants’ consent. The participants for the current study were recruited from the survey sample based on their availability and willingness to participate. They were individuals aged 18 years or older who had lived in the study communities continuously for more than six months. Women who were either pregnant or lactating for less than six months were excluded from the study. While lactating may cause weight loss among mothers, pregnancy is often associated with weight gain. Thus, both physiological states might introduce bias in their body mass index computation.

### Data analysis

The audio tapes of the interviews were transcribed verbatim by the two trained research assistants. To ensure accuracy, the first author and a data auditor proficient in English, Ga, and Twi languages listened to the audio files alongside the transcripts. The transcripts were repeatedly read to get familiar with the data, and the first author developed codes using a thematic approach in Atlas ti.7. An initial coding framework was developed based on the interview guide and by identifying inductive codes based on emerging themes. The coding framework was reviewed by three doctoral candidates experienced in qualitative data analysis. The revised coding framework was further collectively examined by all authors to reconcile conflicting codes.

## Results

### Socio-demographic characteristics of the participants

A total of 44 individuals partook in the focus group discussions; with an average of 5 participants in each group. The sample size was to enable easy moderation and also encourage participation among members. The ages of the participants ranged from 18 to 71 years and the majority of them were males (25). Almost all the participants had attained some level of formal education except five persons who had never been to school.

Additional demographic and socio-economic characteristics of the participants are presented in Table 1.

**Table 1:** Socio-demographic characteristics of the participants.

Characteristics	Number	Percentage
**Sex of respondents**		
Males	25	56.8
Females	19	43.2
**Age**		
18–35	22	50.0
36–71	22	50.0
**Level of education**		
No education	5	11.4
Primary	4	9.1
Middle/JHS	21	47.7
Secondary+	14	31.8
**Marital status**		
Married	24	54.5
Widowed/divorced	8	18.2
Never married	12	27.3
**Employment status**		
Employed	29	65.9
Unemployed	15	30.1
**Body mass index (BMI)**		
Normal weight (18.5 to 24.9 kg/m^2^)	22	50.0
Overweight (25 to 29.9 kg/m^2^)	13	29.5
Obese (≥ 30 kg/m^2^)	9	20.5
**Weight management goal**		
Lose weight	10	22.7
Gain weight	2	4.6
Stay about the same	32	72.7

Source: Field Data, 2016

### Benefits of weight management

The benefits of weight management were discussed under six basic themes. These include: (1) reduces the risk of diseases; (2) encourages physical movements, (3) promotes longer life, (4) prevents tiredness, (5) enables one to wear fashionable clothes and (6) helps maintain clothes for an extended period.

#### A healthy weight reduces the risk of diseases

There was a consensus among the participants that being fat increases one’s risk of diabetes, hypertension, high cholesterol, and knee and waist pains. Therefore, it is beneficial for an individual to attain a healthy weight to prevent such diseases. The young women’s focus group discussion (FGD) noted as follows:

“*Health-wise*, *it is good to manage weight because excess weight is associated with cholesterol*, *blood pressure*, *diabetes*, *and the rest*. *You will therefore prevent all these when you have normal body size” (Young women*, *Ussher Town)*.

“*Being fat is not a good thing because it comes with all sorts of illnesses*” *(Young women*, *James Town)*.

#### Healthy weight encourages physical movement

The participants indicated that being fat restricts physical movement while having a healthy body size encourages physical movements such as walking and going about one’s duties without any restriction. Thus, it is beneficial to manage weight to improve physical movements such as walking. Older men and younger women made the following observations:

“*It is very beneficial to manage your weight*. *When you are of normal weight*, *walking about and going about your normal duties will not become a problem*. *So we think it is good to maintain a normal body weight*. *This will help us go about our normal body duties briskly (Older men*, *James Town)*.

“Bing fat *is not good at all*. *You will have challenges in walking but someone who is of normal weight will easily move about” (Younger women*, *James Town)*.

#### Promotes longer life

The participants shared that managing one’s body weight through exercising, for instance, tends to prolong one’s life. The older men’s group made the following observation:

“*If you can manage your weight*, *you will live longer even though we will all die*. *So exercising to manage our weight will help us live longer” (Younger men*, *James Town)*.

“*When you manage your body weight*, *you can stay healthy and live longer*. *When you engage in exercises*, *you can stay longer even though God is the giver of life*" *(Older men*, *Ussher Town)*.

#### Reduces tiredness

The participants perceived that weight management reduces tiredness when performing an activity. In other words, weight management enhances physical fitness.

“*When you manage your weight and have a normal weight*, *it does not make you get tired quickly when performing an activity*” *(Older women*, *Ussher Town)*.

#### Wear fashionable clothes

The younger women’s group participants indicated that weight management enables a person to wear fashionable clothes, particularly ‘fitting’ (tight) clothes. They explained that managing one’s weight helps lose excess fat and makes one look attractive. In this regard, they can wear fashionable clothes.

“*When you manage your weight*, *you always look attractive*. *But when you gain excess weight with a big stomach*, *you cannot wear the fashionable dress called ’fitting’*. *A big stomach becomes a threat to the dressing*, *so sometimes the weight management gives you a flat stomach to dress easily*. *There are some dresses that you would like to wear to fit your body*, *but when you pick them*, *you realize you cannot wear them when you become fat” (Younger women*, *James Town)*.

### Barriers to weight management

The barriers to weight management were discussed under three main domains: barriers to healthy eating, barriers to engagement in physical activity and social barriers to weight management.

#### Diet-related barriers

The participants reported high cost of fruits and vegetables, fruits and vegetables are not filling, lack of money/poverty, adverse reactions from fruit intake, ignorance about the benefits of fruits and vegetables, and negative perceptions about fruits and vegetables as barriers to healthy eating.

*High cost of fruits and vegetables*. The participants stated that compared to some carbohydrate foods such as kenkey (staple food made from corn meal), fruits and vegetables are expensive. Therefore, members of the community prefer to eat kenkey. This perception was widespread among both males and females.

“*You will see a small apple which costs two Ghana cedis*, *a small piece of watermelon for one Ghana cedi*. *That money can buy kenkey and fish*. *So you will rather eat the kenkey*”*(Older men*, *Ussher Town)*.

“*The community members believe fruits and vegetables are costly*, *so they prefer to buy kenkey and fish instead of fruits*. *Looking at a ball of kenkey*, *which is one cedi equating to pineapple or watermelon at the same cost of one cedi*, *they will rather buy kenkey*” *(Younger women*, *James Town)*.

*Fruits and vegetables do not fill the stomach*. The participants consider fruits and vegetables as expensive; they also expressed that the consumption of fruits and vegetables does not fill the stomach or does not give satisfaction compared to carbohydrate foods. The young women’s focus group discussion (FGD) noted as follows:

“*We prefer food with lots of carbohydrates to fruits*. *Something that can sustain us for longer hours*. *Fruits and vegetables are not foods you can eat to feel satisfied*” *(Younger women*, *Ussher Town)*.

In addition, some participants noted that they are poor and do not have enough money to purchase the foods that will satisfy them in addition to fruits and vegetables. Therefore, they have to choose, so they choose foods that will fill their stomach and carry them through the day.

“*Eating fruits and vegetables require money*, *it is money matter*, *but there is no money*. *We cannot buy heavy food and also buy fruits and vegetables*. *You see*, *it is all due to poverty*. *We have to choose*, *so we want to eat the food which will fill us for long hours*” *(Younger men*, *James Town)*

*Adverse reactions from fruit intake*. Furthermore, some participants indicated that they do not consume some fruits because they usually encounter some adverse effects, especially diarrhoea, when they consume them. Particular reference was made to fruits such as oranges, apples and pineapples.

“*There are people who do not like to eat fruits like oranges and pineapples because they believe that when they eat them*, *they end up having diarrhoea*” *(Older men*, *James Town)*.

In the younger men’s group, it was indicated that most community members do not consume fruits because they are ignorant of the benefits derived from fruits and vegetables.

"*Most of the time*, *it is ignorance*. *In this community, most people do not know the importance of fruits and vegetables to the body*. *So*, *what they will use in buying fruits*, *they prefer to use it to buy alcohol to have an appetite for their meal*” *(Younger men*, *James Town)*.

#### Physical activity-related barriers

The participants noted that lack of time, laziness, stress, and side effects of exercising inhibited their participation in physical activity.

*Lack of time*. The participants stated that occupational activities took away the time which could have been used to engage in physical activity. Specifically, they created the impression that exercise could only be performed at dawn; however, this was the time most people embarked on their economic activities.

“*There are people who wake up at dawn to go to work*. *So they find it difficult to use the same time to exercise*. *Dawn is the time to exercise*, *but they will be working to earn money instead of exercising*. *So it’s all about getting free time" (Older women*, *James Town)*.

“*It is all about getting free time*. *If you are in the home doing nothing*, *you can exercise as much as possible*, *but immediately you get a job*, *you will not have much time anymore*" *(Older women*, *Ussher Town)*.

*Laziness*. Laziness was mentioned as a critical factor limiting participation in physical activity. This was highlighted in the men’s group.

“*Laziness! Laziness!! Laziness!!! That is what hinders a lot of people in this community from exercising*” *(Older men*, *James Town)*.

*Adverse effects of physical activity*. It was indicated in the women’s group that most women encounter some adverse effects when they engage in physical activity. For instance, some feel hungry, too tired, and gain weight when they engage in physical activity. All these side effects prevent them from engaging in physical activity.

“*Some people also say that exercise makes them go hungry that is why they have stopped exercising*” *(Older women*, *Ussher Town)*.

“*Exercise makes some people rather heavy instead of losing some weight*, *so they don’t exercise*” *(Older women*, *Ussher Town)*.

#### Social barriers

Two sub-categories were discernible under this domain–community perceptions about body size, and nutritional value of staple foods.

*Community perceptions about body size*. The participants expressed that being slim or weight loss was not socially acceptable, associated with poverty and diseases, including HIV/AIDs. In addition, slim persons in the community are mocked with derogatory terms such as ’bones’, fried fish’, ’doll’, and ’chingilingi’ (someone extremely slim). Due to these ways of mocking and stigmatisating slim persons, plump individuals do not wish to lose weight. On the other hand, slim persons and those who have normal weight desire to gain weight, and they mostly subscribe to medications. Some participants described the situation in the following statements:

“*There are things in this community that will enable a person to lose weight when you are big*. *However*, *the mockery you will face will deter you from doing so*. *People may say that you have AIDs when they see that you have lost weight*. *Due to that*, *we do not want to lose weight even though some of us are fat*. *When you are slim*, *or of normal weight*, *you would like to gain we`ight due to the mockery you will face*. *So over here*, *people try to gain weight using medicines*” *(Older women*, *Ussher Town)*.

*Some people are even mocked because they are slim*. *Since they (mockers) have gained weight and you are slim*, *you will be teased*. *People will always be talking about the fact that you are too slim*. *Is it that your husband is not able to provide you with money to buy some of the drugs? Don’t you eat before you sleep? Doesn’t your husband allow you to sleep? These are some of the questions that people will ask*. *It will disturb you a lot*. *So*, *if you are given 2 cedi*, *to buy food*, *you will use it to buy the drug*. *So that the next time you get the chance to eat*, *you can take the drug*. *The next time the person sees you*, *she will be shocked*, *and will realize that you are now looking “fine”*. *So that is what is happening*” *(Older women*, *James Town)*

While being slim is mocked and stigmatized, the participants indicated that being fat is cherished and linked with positive attributes, including beauty, respect, wealth, and attractive dressing. Regarding the attractive dressing, participants in the older women’s group reported that some traditional clothing including "Kaba and slit", is better suited to an overweight or obese body size than a slimmer body size. And this stimulates the desire to gain weight.

“*In this community*, *when you grow fat*, *they say you have become fine*. *You have become beautiful*” *(Younger men*, *Ussher Town)*.

“*To them (community members)*, *when you are fat or have a big body size*, *and you put on clothes like kaba and slit*, *it is more beautiful than when you are slim*, *so they prefer to gain weight*” *(Older women*, *James Town)*.

*Nutritional value of community’s staple foods*. The community’s staple foods were also mentioned by some of the participants as a barrier to healthy eating. According to the participants, the community’s staple foods (*Kenkey and banku made from cornmeal*) are high in carbohydrates. Thus, the consumption of such foods increases their risk of gaining weight.

“*Banku and Kenkey*. *That’s all we eat here*. *It is difficult to change*, *and this also makes us to gain weight*” *(Younger women*, *Ussher Town)*.

“*In Ga*, *we have staple foods*, *and these are* “*banku*”, *rice*, *and* “*kenkey*”. *That is all we eat and this also affects our body size*” *(Younger men*, *James Town)*.

### Facilitators of weight management

The participants listed five factors that enable participation in weight management. These include personal decisions, the realisation of positive effects of physical activity, health professionals’ advice, media influence, support from a church, and support from a keep fit club.

#### Personal decision

The participants pointed out that an individual has to decide to initiate weight management personally to achieve one’s aim.

“*The person has to make up his mind to manage his weight*. *It is like a friend asking you to stop wee [marijuana] smoking*. *If you have the willpower*, *you will stop*” *(Younger men*, *James Town)*.

#### Positive effects of physical activity

Also, some of the participants stated that when one sees a reduction in weight after a period of engaging in physical activity, it encourages the individual to further engage in physical activity to manage one’s weight.

“*If an individual is slim*, *one should not desire to become fat*, *and if the person is the obesity type*, *one should do some little exercise*. *But if you do the exercise and still are of the same size*, *you don’t have to worry about yourself*. *You are encouraged to continue exercising when you see a reduction in weight*” *(Younger women*, *Ussher Town)*

The participants also noted that health professionals’ advice to eat healthy foods promotes weight management.

“*Unless it is written from the doctors to eat healthy to manage weight*, *they will not do it*. *It is when doctors advise before they will try to follow the doctors’ advice*” *(Younger men*, *Ussher Town)*.

In addition, the participants reported that media influence and support from a church or a keep fit club motivate engagement in weight management behaviours These factors were mentioned by the women’s group only. For instance, they reported that in contemporary times, the media show personalities with normal body sizes, which influences them to exercise to attain such body size.

“*Nowadays*, *the media have influenced people*. *When they see the nice body shape on TV then we the ladies*, *we also want to have such body shape*, *so we try to exercise*” *(Older women*, *Ussher Town)*.

Finally, association with churches and keep fit clubs encouraged people to exercise—the participants explained that these organisations occasionally organise health talks and physical activities.

“*For us*, *our church sometimes invites doctors and trainers around who help us engage in exercise*. *So*, *once in a while*, *we do exercise*. *They also talk to us about eating good diets*” *(Younger women*, *James Town)*.

“*Both men and women go for jogging together*. *Sometimes when the men wake up early*, *they call the women*, *which helps because going jogging by oneself may be challenging*” *(Older women*, *James Town)*.

## Discussion

This study provides in-depth insight into weight management regarding benefits, barriers, and facilitators in poor urban Accra, Ghana. The results showed that the participants are knowledgeable about the benefits of attaining healthy body weight. For instance, they perceived obesity as detrimental to health as it increases an individual’s risk of non-communicable diseases, restricts physical movement, and reduces one’s life span. In addition, attaining a healthy body weight was considered beneficial because it reduces risks of diseases, encourages physical movements, and promotes longer life. These findings are similar to those reported in other obesity studies such as Okop et al.’s [20], which found that participants generally believed that obesity could lead to health conditions such as heart attack, stroke, diabetes, and hypertension. Notably, the participants’ understanding of the health effects of obesity, to some extent, overlaps with the biomedical explanation. Obesity is a known risk factor for chronic non-communicable diseases like cardiovascular diseases and diabetes. It also reduces the quality of life and leads to premature death [7].

Although the participants knew the benefits of weight management, the knowledge seems to be overshadowed by some barriers to weight management. One key barrier found in this study is community perceptions about body size: while weight loss or being slim is mocked and stigmatized, being fat is cherished. The concerns about being stigmatized for weight loss compel individuals to use non-prescribed medications to stimulate weight gain. Similar findings have been reported in other studies in sub-Saharan Africa, which indicate that most overweight and obese women do not want to lose weight because of the stigma attached to being slim [15, 20, 21]. The finding highlights the vital role social processes play in shaping people’s body size preferences and related behaviours [22]. The findings from the present study have programmatic implications in terms of health education strategies and messaging. There is the need to campaign against and undermine the traditional notion of associating fat with wealth, beauty, respect, and good health at the community level.

The cost of healthy foods was also reported as another key factor that hindered healthy eating. The study communities are predominantly a Ga society whose staple foods (kenkey and banku- local foods made from fermented corn dough) are relatively less costly than healthy foods such as fruits and vegetables. This disparity in cost prevents individuals from having a healthy eating lifestyle. The limited financial resource may explain the perception that fruits and vegetables are expensive in urban poor settings. In an urban poor environment in South Africa, a study conducted by Okop et al. [23] reported the lowest purchase and daily consumption of fruits and vegetables in rural and poor urban areas is due to inadequate purchasing power. However, high proportions of the participants living in poor urban communities had purchased sugary drinks daily/weekly because they were cheaper and affordable. Also, Layede and Adeoye’s [24] study in Nigeria among tertiary education students found that the cost of fruits and vegetables created a barrier to healthy eating. Comparable to other studies [16, 20], the participants in this study referred to poverty as a barrier to healthy eating. The expression of the high cost of fruits and vegetables coupled with the issue of poverty demonstrates the extent to which socio-economic status influences health inequality, particularly in low resource settings.

Some studies have reported a positive association between socio-economic status and health [25, 26]. The poor tend to have limited economic resources, limiting their ability to afford healthy foods and lifestyles [27]. However, poor agricultural households may have access to fresh vegetables and fruits from their farm. In contrast, the poor in urban settings may not have such opportunities, especially in poor, overcrowded communities with no open gardening spaces. In Kenya, through the National Youth Service (NYS), the government championed an unusual form of urban farming: sack gardening in Kibera. This involves growing various crops and vegetables on the top and sides of large burlap sacks filled with soil, small stones, and manure. This approach is seen as a cheap and healthy solution to food insecurity in Nairobi’s largest urban poor settlement with limited space [28]. This strategy could be adopted in the study communities to increase access and consumption of fresh fruits and vegetables.

Another main findings is that some participants perceive fruits and vegetables as not filling, unlike the community’s staple foods. Thus, one may need to consume more fruits and vegetables to satisfy one’s hunger. Consequently, much capital is required to purchase more fruits and vegetables. Participants thus perceive that the consumption of fruits and vegetables is expensive since they do not have enough money to access them in higher quantities. Therefore, such participants are compelled to make choices, so they choose foods that will satisfy and carry them throughout the day. Research has demonstrated that poverty influences choice of food. That is, poverty limits an individual’s purchasing power and thus narrows one’s diet pattern. Having limited income and social agencies places many individuals in a situation with no choice of a healthy diet. Therefore, they may eat anything that comes their way to survive [23]. Consuming fruits and vegetables serve as preventive health measures. Thus, participants should make every effort to consume the number of fruits and vegetables their available capital can afford to promote healthy living. In addition, health practitioners should embark on community-level education on the benefits of fruits and vegetables and healthy eating, coupled with health promotion activities such as procuring and preparing fruits and vegetables with little cost.

The findings also indicate that laziness and lack of time undermine individuals’ efforts to participate in physical activity. These findings are consistent with previous studies that reported factors including lack of time and laziness as barriers to weight management [10, 13]. For change to occur, the individual must be prepared to initiate it. In the present study, personal decisions emerged as one of the key facilitators of weight management. This suggests that personal motivation and self-restraint should be considered in an intervention concerning weight management.

Notably, the finding from this study which indicates that physical activity could only be performed at dawn and that engagement in economic activities denies people the opportunity to engage in physical activity, is thought-provoking. There is inadequate knowledge that physical activity can be performed at other times and, therefore, calls for policy intervention. Walking, for example, at least 30 minutes three times per week is recommended as a minimum requirement to attain better blood circulation [29]. Community-level education on the components and recommended physical activity levels should be instigated.

Another primary finding from this study is the role of health practitioners, media, and social organisations, including church and keep fit clubs, in motivating engagement in weight management behaviours through health messages. These findings corroborate those of other studies, including that of Hammarström et al. [30] in Sweden, which provided intervention for women to lose weight. The support received from the project to eat healthy food during group meetings and interactions with nurses and dieticians enabled them to eat healthy foods. In Pakistan, advertisements for thin body size as the ideal body size influenced the desire to lose weight [31]. In Ghana, the media can use some celebrities to champion weight management, just as some celebrities have been used for advertisements on foods such as Milo (cocoa drink), tin tomatoes, vitamilk (soymilk drink), alcoholic beverages, etc. Importantly, female members of parliament should be encouraged to get involved in messages or speak on these issues in the media.

Also, some studies have demonstrated that association with a religious group positively influences health behaviours. This is because the church members receive support from co-church members to practice a healthy lifestyle [32]. Based on the findings, promoting and educating social and community health clubs (e.g. Keep fit clubs, churches) on issues related to weight management is essential for effective interventions. In addition, attention should focus on the ill effects of inappropriate weight management practices, particularly the use of non-prescribed medication to gain weight. A collaboration between health practitioners and these social and religious organisations is essential for effective interventions.

## Conclusion

This study showed that factors, including the cost of healthy foods, poverty, and the perception that fruits and vegetables are not filling in the stomach are the main factors that hindered healthy eating among the participants. Also, laziness and lack of time to exercise prevented individuals from engaging in physical activity. Furthermore, it was evident from this study that being fat or slim were both not perceived as healthy features by the participants. However, social representations of overweight/obesity and weight loss influenced individuals’ desire for larger body size. This affected the choice of weight management behaviours, particularly medications to gain weight. This study provides evidence that can be used to tailor interventions for weight management in this population; and may be extended to the rest of Ghana since the social perceptions of weight, weight gain, and being slim, as well as the factors associated with them, are about the same among other ethnic groups in Ghana [22].

## Supporting information

S1 File Interview guide on perceptions of weight management. (DOCX)

S2 File Data on perceptions of weight management. (DOCX)

## References

[1] Malik VS, Willet WC, Hu FB. Nearly a decade on—trends, risk factors, and policy implications in global obesity. Nat Rev Endocrinol. 2020; 16, 615–616. 10.1038/s41574-020-00411-y 32873971 PMC7461756

[2] Lartey ST, Magnussen CG, Si L, Boateng GO, de Graaff B, Biritwum RB, et al. Rapidly increasing prevalence of overweight and obesity in older Ghanaian adults from 2007–2015: Evidence from WHO-SAGE Waves 1 & 2. PLoS ONE. 2019: 14(8): e0215045. 10.1371/journal.pone.0215045 31425568 PMC6699701

[3] Qasim A, Turcotte M, de Souza RJ, Samaan MC, Champredon D, Dushoff J, et al. On the origin of obesity: identifying the biological, environmental and cultural drivers of genetic risk among human populations. Obesity Reviews. 2017: 19, 121–149. doi: 10.1111/obr.12625 29144594

[4] Ghana Statistical Service (GSS), Ghana Health Service (GHS), ICF International. Ghana demographic and health survey 2014. Rockville, Maryland: GSS, GHS, and ICF International; 2015.

[5] Agyemang C, Boatemaa S, Frempong GA, De-Graft Aikins A. Obesity in Sub-Saharan Africa. In Ahima RS (Ed.) Metabolic Syndrome. A Comprehensive Textbook, 2016. 41–53. Springer International Publishing, Switzerland, Cham.

[6] Seidell JC, Halberstadt J. The Global Burden of Obesity and the Challenges of Prevention. Annals of Nutrition Metabolism. 2015: 66(suppl 2):7–12. doi: 10.1159/000375143 26045323

[7] Tremmel M, Gerdtham U-G, Nilsson PM, Saha S. Economic Burden of Obesity: A Systematic Literature Review. International Journal of Environmental Research and Public Health. 2017; 14, 435. doi: 10.3390/ijerph14040435 28422077 PMC5409636

[8] Soleymani T, Daniel S, Garvey WT. Weight maintenance: challenges, tools and strategies for primary care physicians. Obesity reviews. 2016: 17, 81–93. doi: 10.1111/obr.12322 26490059 PMC4715703

[9] Obino KFM, Pereira C.A, Caron-Lienert RS. Coaching and barriers to weight loss: an integrative review. Diabetes, Metabolic Syndrome and Obesity:Targets and Therapy. 2017:10.1–11 doi: 10.2147/DMSO.S113874 28096687 PMC5207339

[10] Aziz NSA, Zaki NAM, Nor NSM, Ambak R, Man CS. Perspective on Obesity Problems and Associated Factors to Reduce Weight among Overweight and Obese Housewives: A Qualitative Study. Journal of Women’s Health, Issues and Care, 2016.

[11] Woodruff RC, Schauer GL, Addison AR, Gehlot A, Kegler MC. Barriers to weight loss among community health centre patients: qualitative insights from primary care providers. BMC Obesity. 2016: 3:43. doi: 10.1186/s40608-016-0123-3 27785364 PMC5073457

[12] Metzgar CJ, Preston AG, Miller DL, Nickols-Richardson SM (2015). Facilitators and barriers to weight loss and weight loss maintenance: a qualitative exploration. Journal of Human Nutrition and Dietetics, 28(6), 593–603. doi: 10.1111/jhn.12273 25231461

[13] Abolhassani S, Doosti Irani M, Sarrafzadegan N, Rabiei K, Shahrokhi S, Pourmoghaddas Z, et al. Barriers and facilitators of weight management in overweight and obese people: Qualitative findings of TABASSOM project. Iranian Journal of Nursing and Midwifery Research. 2012; 17(3). 23833613 PMC3696212

[14] Walcott-McQuigg JA. Weight control behaviour and women: a cross-cultural perspective. Journal of International Women’s Studies. 2005. 7(2), 152–168.

[15] Draper CE, Davidowitz KJ, Goedecke JH. Perceptions relating to body size, weight loss and weight-loss interventions in black South African women: a qualitative study. Public Health Nutrition. 2015; 1–9. doi: 10.1017/S1368980015001688 26006784 PMC10270930

[16] Accra Metropolitan Assembly–UN Habitat (AMA-UN Habitat): Participatory slum upgrading and prevention: millennium city of Accra, Ghana. Accra: AMA; 2011.

[17] Afrifa–Anane E, Agyemang C, Codjoe SNA, Gbenga Ogedegbe G, de-Graft Aikins A. The association of physical activity, body mass index and the blood pressure levels among urban poor youth in Accra, Ghana. BMC Public Health. 2015; 15:269. doi: 10.1186/s12889-015-1546-3 25881047 PMC4376361

[18] de-Graft Aikins A, Kushitor M, Koram K, Gyamfi S, Gbenga Ogedegbe G. Chronic noncommunicable diseases and the challenge of universal health coverage: insights from community-based cardiovascular disease research in urban poor communities in Accra, Ghana. BMC Public Health. 2014;14(S2):S3. http://www.biomedcentral.com/1471-2458/14/S2/S3 10.1186/1471-2458-14-S2-S3 PMC4120153 25082497

[19] World Health Organization (WHO). Obesity: preventing and managing the global epidemic report. Geneva (Switzerland): World Health Organization. 2004 11234459

[20] Okop KJ, Mukumbang FC, Mathole T, Levitt N, Puoane T. Perceptions of body size, obesity threat and the willingness to lose weight among black South African adults: a qualitative study. BMC Public Health. 2016; 16:365. doi: 10.1186/s12889-016-3028-7 27129700 PMC4850665

[21] Matoti-Mvalo T, Puoane TB. Perceptions of body size and its association with HIV/AIDS. South African Journal of Clinical Nutrition. 2011 24(1). Retrieved from http://www.ajol.info/index.php/sajcn/article/viewFile/65390/53082.

[22] Tuoyire DA, Kumi-Kyereme A, Doku DT, Amo-Adjei K. Perceived ideal body size of Ghanaian women: “Not too skinny, but not too fat”, Women & Health. 2017. doi: 10.1080/03630242.2017.1321607 28426342

[23] Okop KJ, Ndayi K, Tsolekile L, Sanders D Puoane T. Low intake of commonly available fruits and vegetables in socio-economically disadvantaged communities of South Africa: influence of affordability and sugary drinks intake. BMC public health, 2019; 19(1), pp.1–14. 10.1186/s12889-019-7254-7 31299939 PMC6626349

[24] Layede AA, Adeoye IB. Fruit and vegetable consumption among students of tertiary institution in Oyo state. Int Inf Syst Agric Sci Technol. 2014;(6):3–8.

[25] Sui J, Giskes K, Turrell G. Socio-economic differences in weight-control behaviours and barriers to weight control. Public Health Nutrition: 2011;14(10), 1768–1778. doi: 10.1017/S1368980011000644 21557867

[26] Lago S, Cantarero D, Rivera B, Pascual M, Blázquez-Fernández C, Casal B, et al. Socio-economic status, health inequalities and non-communicable diseases: a systematic review. Journal of Public Health. 2017:1–14. doi: 10.1007/s10389-017-0850-z 29416959 PMC5794817

[27] Friel S, Chopra M, Satcher D. Unequal weight: equity oriented policy responses to the global obesity epidemic. British Medical Journal, 2007; 7632, 1241. 10.1136/bmj.39377.622882.47 PMC2137064 18079548

[28] Gallaher CM, WinklerPrins AMGA, Njenga M, Karanja NK. Creating space: Sack gardening as a livelihood strategy in the Kibera slums of Nairobi, Kenya. Journal of Agriculture, Food Systems, and Community Development, 2015; 5(2), 155–173. 10.5304/jafscd.2015.052.006

[29] de Munter J. Physical activity in a multi-ethnic population: measure and associations with cardiovascular health and contextual factors. Amsterdam, The Netherlands. Ipskamp Drukkers BV, Enschede. 2012.

[30] Hammarström A, Wiklund AF, Lindah B, Larsson C, Ahlgren C. Experiences of barriers and facilitators to weight-loss in a diet intervention—a qualitative study of women in Northern Sweden. BMC Women’s Health. 2014; 14:59. doi: 10.1186/1472-6874-14-59 24739099 PMC3998240

[31] Jaffari MW, Zaman HMF, Hayat Q, Munir MH, Saleem S. Do media develop eating disorders: a study with reference to young females of Pakistan. European Journal of Scientific Research. 2011; 57(1), 29–46.

[32] Krause N, Shaw B, Liang J. Social relationships in religious institutions and healthy lifestyles. Health Education & Behavior. 2011; 38(1), 25–38. doi: 10.1177/1090198110370281 21169477 PMC3043376

