# Supplementary material for: Barriers and facilitators of weight management: Perspectives of the urban poor in Accra, Ghana
Source: PLoS One. 2022 Aug 8;17(8):e0272274. doi: 10.1371/journal.pone.0272274 (PMC9359579; doi:10.1371/journal.pone.0272274)
Supplement: S1 File — (DOCX) [file pone.0272274.s001.docx]

**Interview Guide for focus group discussion**

In this section, the questions focus on your perceptions about the factors that influence weight management in this community.

1. Tell us a little bit about yourself

Probes: age, educational status, occupation, marital status etc

1. Which picture do you most look like right now?

Note: Show body image cards to respondents and prompt them to describe their current body size using the figural stimuli.

1. During the past 12 months, what have you been trying to do about your body weight?

Probes: lose weight, gain weight, stay about the same

1. Do you think the community members would consider changing their body size (weight management)? *Probes:* If yes, would they want to put on some more weight, lose weight or stay same?
2. What strategies do you think those who would consider changing their body size will adopt? *Probes:* physical activity, diet, medication. Explain your answer.
3. Are there barriers to weight management in this community? Or, are there factors that will prevent community members from changing their body size?

-Probe for perceptions about ideal body size, cultural practices (intake of certain foods during postpartum periods, staple foods).

-Perceptions about physical activity and healthy food such as fruits and vegetables

-Do you think the media, church, mosque and other social organisation influence weight management? Explain

1. Are there factors in this community that will encourage or enable an individual to initiate weight management?
2. Could you suggest recommendations for weight management in this community?
