# Supplementary material for: Barriers and facilitators of weight management: Perspectives of the urban poor in Accra, Ghana
Source: PLoS One. 2022 Aug 8;17(8):e0272274. doi: 10.1371/journal.pone.0272274 (PMC9359579; doi:10.1371/journal.pone.0272274)
Supplement: S2 File — (DOCX) [file pone.0272274.s002.docx]

Appendix D: Focus group discussions (FGDs) coding frame

| **Global theme** | **Organising theme** | **Sub-organising theme** | **Basic Theme** | **Description** | **Sample Quote(s)** |
| --- | --- | --- | --- | --- | --- |
| Perceptions of weight management | Barriers to weight management | Individual level barriers | High cost of fruits and vegetables | High cost of fruits prevents individuals from choosing fruits and vegetables as a weight management behaviour | ***I:*** *What do you think will be the hindrances to intake of fruits and vegetables to manage weight?*  ***R:*** *The kenkey is what does that. Probably the person is only having 2 Ghana cedis. You know that you can buy kenkey and fish with that amount of money. However, the cost of the vegetables is expensive, and she might have to spend extra money to get that” (Older women, James Town****).***  *“You will see a small apple costing 2 Ghana cedis, a small piece of watermelon for 1 Ghana cedis. That money can buy kenkey and fish. So you will rather eat the kenkey” (older Men, Ussher Town****).***  *“The community members believe fruits and vegetables are costly, so they prefer to buy kenkey and fish instead of fruits. Looking at a ball of kenkey, which is one cedi equating to pineapple or watermelon at the same cost of one cedi, they will rather buy kenkey” (Younger women, James Town).*  *“Some of the fruits are very expensive. The rotten ones are cheaper. However, they are also not good for our health because a lot of house flies would have fed on it by then” (Older men, James Town).* |
|  |  |  | Adverse reactions from fruit intake | Diarrhoea reactions encountered from the intake of some fruits prevent individuals from eating fruits | *“There are people who do not like to eat fruits like oranges and pineapples because they believe that when they eat them, they end up having diarrhoea” (Older men, James Town).*  *“For me when I take in oranges my stomach hurts so I don’t like them. It is the same with apple so I don’t normally eat fruits” (Older women, James Town).* |
|  |  |  | Fruits and vegetables are not filling | Consumption of fruits and vegetables do not give satisfaction | *“He will tell you it will not satisfy him, rather he will buy plenty spaghetti for one cedi and eat. Afterwards, he will drink pure water to survive the day” (Younger men, Ussher Town.)*  *“we prefer food with lots of carbohydrates to fruits. Something that can sustain me for long hours” (Younger women, Usher Town).*  *“Eating fruits and vegetables require money, it is money matter, but there is no money. We cannot buy heavy food and also buy fruits and vegetables. You see, it is all due to poverty. We have to choose, so we want to eat the food which will fill us for long hours” (Younger men, James Town)* |
|  |  |  | Ignorance of benefits of fruits and vegetables | Ignorance about the importance of fruits and vegetables is a barrier to weight management | *“Most of the time, it is ignorance. They do not know the importance of fruit and vegetables. So, what they will use in buying fruits, they prefer to use it to buy alcohol so that they will have an appetite for their meal”. (Younger men, James Town)* |
|  |  |  | Poverty | Individuals lack money to eat healthy foods | *“Weight management helps, but here it will not help me because with GHC 10.00 the four of us can buy banku Ghc. 5.00 so which one will we use to buy the fruits? You see it is all due to poverty (Young women, Ussher Town).*  *“They do not have money to go to a restaurant to buy salad” (younger men, James Town).*  *“Most of the time, it happens when you do not have anything proper doing. You will usually stress yourself up in search of a job. Since you do not have enough money, you will not be able to buy any healthy food. Some will even have to sleep with men before they get what to eat. So I believe if you have money, you will be able to eat healthy”. (Older Men, Ussher Town)* |
|  |  |  | Perceptions of fruits safety | ‘Perceptions  about fruits and vegetables prevent their consumption | *“Some people are of the view that most of the fruits are not good organically because of the fertilizer being used. So they end up contracting illness after consumption” (Younger men, James Town)* |
|  |  | Exercise related barriers | Laziness | Laziness hinder individuals from exercising | *: “For example, as a footballer if you feel lazy to play soccer you will not be able to do it. So, if you avoid laziness and decide to lose weight within a few months you can do it” (Younger men, James Town).*  *“Laziness! Laziness!! Laziness!!! That is what hinders a lot of people in this community from exercising” (Older men, James Town).* |
|  |  |  | Lack of Time | Individuals do not have the time to exercise | *There are people who wake up at dawn to go to work. So they find it difficult to use the same time to exercise. Dawn is the time to exercise, but they will be working to earn money instead of exercising. So it's all about getting free time" (Older women, James Town).*  *“It is all about getting free time. If you are in the home doing nothing, you can exercise as much as possible, but immediately you get a job, you will not have much time anymore" (Older women, Ussher Town).* |
|  |  |  | Side effects of exercise | Side effects of exercising prevent individuals from exercising | *“Some people also say that exercise makes them go hungry that is why they have stopped exercising” (Older women, Ussher Town).*  *"Exercise makes some people rather heavy instead of losing some weight, so they don’t exercise” (Older women, Ussher Town).* |
|  |  | Community level barriers | Nutritional value of staple foods | The community staples are carbohydrate in nutrient | *"In Ga, we have staple foods, and that is "banku", rice, and "kenkey". That is all we eat and this also affects our body size” (Younger men, James Town).*  *“Banku and Kenkey. That’s all we eat here. It is difficult to change, and this also makes us to gain weight” (Younger women, Ussher Town).*  “For the Gas, because of the kenkey we eat, we are plump” (Older men, Ussher Town) |
|  |  |  | Community perception about body size | |  |
|  |  |  | Stigmatisation of slimness | Slimness is mocked and stigmatised with illness, poverty | *"There are things in this community that will enable a person to lose weight when you are big. However, the mockery you will face will deter you from doing so. People may say that you have AIDs when they see that you have lost weight. Due to that, we do not want to lose weight even though some of us are fat. When you are slim, or of normal weight, you would like to gain weight due to the mockery you will face. So over here, people try to gain weight using medicines" (Older women, Ussher Town).*  “*Slimness is mocked. That is why most people here go in for the medicines. Someone can even ask you to look at yourself to see how dry you look. When that happens, you get worried, so you look for a way to gain weight. If one has money, the person will get one of the medicines to gain weight” (Younger women, James Town).*  *Some people are even mocked because they are slim. Since they (mockers) have gained weight and you are slim, you will be teased. People will always be talking about the fact that you are too slim. Is it that your husband is not able to provide you with money to buy some of the drugs? Don’t you eat before you sleep? Doesn’t your husband allow you to sleep? These are some of the questions that will ask. It will disturb you a lot. So, if you are given 2 cedi, to buy food, you will use it to buy the drug. So that the next time you get the chance to eat, you can take the drug. The next time the person sees you, she will be shocked, and will realize that you are now looking “fine”. So that is what is happening” (Older women, James Town)* |
|  |  |  | Fatness is cherished | Fatness is associated with beauty, health, respect and wealth | *“In this community, when you grow fat, they say you have become fine. You have become beautiful” (Younger men, Ussher Town).*  *“When you grow fat naturally, it is beautiful” ( Younger women, James Town).*  *“To them (community members), when you are fat or have a big body size, and you put on clothes like kaba and slit, it is more beautiful than when you are slim, so they prefer to gain weight” (Older women, James Town).*  “*The perception that fat people are rich is killing us in this community. Therefore, people are doing all they can to gain weight. Of course! Fatness is being sold on the market now. You just drink a lot of beer, eat a lot of food, and drink more beer. Then they grow big” (Older men, Ussher Town)* |
|  | Benefits of weight management |  | Reduces risk of diseases | Having a normal weight reduces an individual’s risk to contract diseases | “*Health-wise, it is good to manage weight because excess weight is associated with cholesterol, blood pressure, diabetes, and the rest. You will therefore prevent all these when you have normal body size” (Young women, Ussher Town).*  *“Being fat is not a good thing because it comes with all sorts of illnesses” (Young women, James Town).*  *“They end up with various illnesses. So for me, I am not in support of anything like that. My mother is very big. As she got older, and has been feeling sick, I have been finding it very difficult to clean her up. So I was finding it difficult to get close to her because I do not find it easy at all, to even lift one arm of hers. I do not think being fat is a good thing at all” (older men, Ussher Town).* |
|  |  |  | Encourages physical movement | Having a normal weight encourages physical movement | “*It is very beneficial to manage your weight. When you are of normal weight, walking about and going about your normal duties will not become a problem. So we think it is good to maintain a normal body weight. This will help us go about our normal body duties briskly (Older men, James Town).*  “*Fatness is not good at all. You will have challenges in walking but someone who is of normal weight will easily move about” (Younger women, James Town).*  *“What my brother is saying is very true. However, those who are slim are able to go out or walk without issues compared to those who are fat. The slim ones do not have any difficulty in working. They are able to briskly go out and do any work due to their stature. However, the fat ones mostly do not want to walk about due to their body size” (Older men, Ussher Town)* |
|  |  |  | Promotes longer life | Weight management enables an individual to live longer | “*If you can manage your weight, you will live longer even though we will all die. So exercising to manage our weight will help us live longer” (Younger men, James Town).*  *“When you manage your body weight, you can stay healthy and live longer. When you engage in exercises; you can stay longer even though God is the giver of life" (Older men, Ussher Town).* |
|  |  |  | Reduces Tiredness |  | *“When you manage your weight and have a normal weight, it does not make you get tired quickly when performing an activity” (Older women, Ussher Town).*  *“You easily get tired when you are overweight. You walk for sometime, and you are tired. Also, when you gain weight, you are not able to go everywhere because you easily get tired. So it is better for you to lose weight than to gain weight” (Older women, James Town)* |
|  |  |  | Fashionable clothes | Weight management makes dressing fashionable | “*When you manage your weight, you always look attractive. But when you gain excess weight with a big stomach, you cannot wear the fashionable dress called 'fitting'. A big stomach becomes a threat to the dressing, so sometimes the weight management gives you a flat stomach to dress easily. There are some dresses that you would like to wear to fit your body, but when you pick them, you realize you cannot wear them when you become fat” (Younger women, James Town).* |
|  |  | Facilitators of weight management | Personal decision | An individual has to personally decide to exercise | *"The person has to make up his mind to manage his weight. It is like a friend asking you to stop wee [marijuana] smoking. If you have the willpower, you will stop" (Younger men, James Town).* |
|  |  |  | Positive effects of physical activity |  | "If an individual is slim, one should not desire fatness, and if the person is the obesity type, one should do some little exercise. But if you do the exercise and still are of the same size, you don't have to worry about yourself. You are encouraged to continue exercising when you see a reduction in weight" (Younger women, Ussher Town) |
|  |  |  | Support from church | Support received from church enables one to exercise | *“For us, our church sometimes invites doctors and trainers around who help us engage in exercise. So, once in a while, we do exercise. They also talk to us about eating good diets” (Younger women, James Town).* |
|  |  |  | Media influence | The media influence people to exercise | *“Nowadays, the media have influenced people. When they see the nice body shape on TV then we the ladies, we also want to have such body shape, so we try to exercise” (Older women, Ussher Town).* |
|  |  |  | Health professionals’ advice | Advice from a doctor to eat healthy | *“Unless it is written from the doctors to eat healthy to manage weight, they will not do it. It is when doctors advise before they will try to follow the doctors’ advice” (Younger men, Ussher Town).* |
|  |  |  | Keep fit club’s support | Support from keep fit club to exercise | *“Both men and women go for jogging together. Sometimes when the men wake up early, they call on the women, which helps because going jogging by oneself may be challenging” (Older women, James Town).* |
